# Endophytic fungi-assisted biological synthesis of zinc oxide nanoparticles using gamma-rays for promising antibacterial and antibiofilm potential against some gram-positive bacteria

**DOI:** 10.1186/s12934-026-02993-0

**Published:** 2026-04-20

**Authors:** Sobhy S. Abdel-Fatah, Nasser H. Mohammad, Gharieb S. El-Sayyad

**Affiliations:** 1 Drug Microbiology Lab., Drug Radiation Research Department, National Center for Radiation Research and Technology (NCRRT), Egyptian Atomic Energy Authority (EAEA), Cairo, Egypt; 2 Radiation Microbiology Department, National Center for Radiation Research and Technology (NCRRT), Egyptian Atomic Energy Authority (EAEA), Cairo, Egypt

**Keywords:** Gamma rays, Endophytic fungi, *Aspergillus tamarii*, Low-cost alternatives, Zinc oxide nanoparticles, Antimicrobial activity

## Abstract

**Introduction:**

Endophytic fungi of medicinal plants have gained recognition as probable sources of bioactive metabolites. In this study, sixteen endophytic fungi were isolated from basil (*Ocimum basilicum*) and screened for antibacterial activity using the agar well diffusion method. The most potent isolate was molecularly identified as *Aspergillus tamarii (A. tamarii)* by the ITS rDNA sequencing method, and the sequence was submitted to GenBank (accession no. PX474848.1). Then the strain was cultivated in different media to reduce production costs and enhance antibacterial metabolite production, including potato peel and *Portulaca oleracea* (*P. oleracea*) as low-cost alternatives to potato extract. The *P. oleracea*-based medium showed the highest antibacterial activity, with inhibition zones of 20.0 mm against *Bacillus subtilis* (*B. subtilis*) and 23.0 mm against *Staphylococcus aureus (S. aureus)*. For the green synthesis of zinc oxide nanoparticles (ZnO NPs), the fungal extract was mixed with an equal volume of 1.0 mM zinc nitrate hexahydrate and exposed to gamma irradiation (20 kGy). Characterization by UV–Vis. spectroscopy, DLS, XRD, SEM, and HRTEM confirmed the formation of ZnO NPs. The synthesized nanoparticles exhibited spherical and rod-shaped morphology and an average size of 70.34 nm. Antibacterial activity was evaluated using the agar well diffusion assay and the broth microdilution method for MIC determination. The biosynthesized ZnO NPs showed inhibition zones of 26.0 ± 0.30 mm against *B. subtilis* and 27.0 ± 0.13 mm against *S. aureus*, with MIC values of 31.25 µg/mL. Antibiofilm activity, assessed by the crystal violet tube method, showed inhibition rates of 50.32 and 55.33% against *B. subtilis* and *S. aureus*, respectively, while protein leakage analysis using the Bradford assay indicated increased membrane permeability in treated bacterial cells. Overall, the bioactive metabolites and the biosynthesized ZnO NPs showed promising antibacterial and antibiofilm activities, possibly linked to increased membrane permeability, suggesting their potential use in food and medical applications.

**Aim:**

The objective of this study was to create a green synthesis of ZnO NPs using bioactive chemicals produced from endophytic fungi and gamma irradiation, as well as a cost-effective approach for producing bioactive metabolites from these fungi.

**Impact statement:**

The current study presents a cost-effective and environmentally friendly process for producing bioactive metabolites from endophytic fungi and biosynthesizing ZnO NPs using gamma irradiation and endophytic fungal bioactive metabolites. The biosynthesized ZnO NPs present viable substitutes for traditional antibacterial agents, especially in food and medicinal applications that target gram-positive bacteria that are resistant to drugs.

**Supplementary Information:**

The online version contains supplementary material available at 10.1186/s12934-026-02993-0.

## Introduction

These days, a number of bacteria that are resistant to antibiotics have emerged as a result of antibiotic abuse [1]. Experts predict that several antimicrobial-resistant bacterial diseases will kill roughly ten million people annually by 2050 [2]. As a result, a lot of research has concentrated on utilizing nanoparticles to create a novel, environmentally friendly antibacterial agent [3]. Among the used nanoparticles, metal oxide nanoparticles have attracted a lot of attention [4].

Among the various metal oxide nanoparticles, zinc oxide nanoparticles (ZnO NPs) are one of the most promising antibacterial agents [5]. Zinc oxide is generally regarded as a safe "GRAS" substance, according to the FDA [6]. ZnO NPs have been used in a wide range of commercial products and applications, including biomedicine [7], food safety paint [8], cosmetics [9], catalysis [10], agriculture [11], and coating [12], due to their distinct chemical, physical, and optical properties [13], low toxicity [14], low cost [15], and biodegradability [16].

Chemicals used to reduce agents in chemical and physical methods are poisonous [17]. It has been shown that the process of making nanoparticles toxic also makes the nanoparticles themselves more poisonous [18]. This could limit the use of NPs in food and medical applications and cause environmental pollution [19].

Biogenic ZnO NP synthesis has many advantages over chemical and physical methods [20]. Microorganisms and plants have metabolites that help keep biogenic synthesis stable and reduce the amount of energy needed [21], and this makes the process safe for the environment. Microbial synthesis of ZnO NPs has become one of the most promising biogenic processes [22, 23]. It has many benefits, such as being safe for the environment, biodegradable, and biocompatible [24].

Literature review strongly recommends the fungus’s extracellular synthesis of ZnO NPs as an economical downstream process for large-scale manufacturing [25–27]. Due to their superior metal tolerance and capacity for metal bioaccumulation, fungi are used in the manufacture of nanoparticles rather than bacteria [28]. There are extracellular myco-materials like proteins and polysaccharides that help make ZnO NPs by acting as reducing agents [23, 29].

Gamma radiation is getting more attention in the synthesis of nanoparticles [30, 31]. This is because it is a simple, green, and environmentally friendly process that radiolysis an aqueous solution and effectively reduces metal ions [32, 33].

*P. oleracea* L., commonly called purslane, is a succulent summer annual plant that is extensively distributed worldwide and is the ninth most common in the world due to its resistance to heat, drought, and salt [34]. Many researchers have reported that purslane has a significant nutritional content of carbohydrates, proteins, essential minerals, and bioactive compounds that can sustain microbial growth and metabolite production [35]. It contains approximately 2% protein and about 3% carbohydrates, along with essential minerals such as potassium (approximately 494 mg/100 g), calcium (about 65 mg/100 g), and magnesium (around 68 mg/100 g) [36].

In addition, purslane is rich in vitamins and phytochemicals, including phenolic compounds and α-linolenic acid, which may influence microbial metabolic pathways [37]. The presence of these nutrients and bioactive constituents suggests that *P. oleracea* could provide alternative beneficial nutrition that may promote fungal growth and potentially modulate secondary metabolite biosynthesis during fermentation. Potato peels represent a carbohydrate-rich agro-industrial by-product with considerable biotechnological potential. Total carbohydrate content ranges between approximately 46 and 88 g/100 g dry weight (DW) [38]. In addition, potato peel contains approximately 30, 18, 20, 1, and 6% of dry weight, non-starch polysaccharides, protein, lignin, lipids, and ash, respectively [39].

Although ZnO NPs have been widely investigated for their antibacterial activity, establishing a cost-effective fungal-based system for their extracellular biosynthesis remains limited. Therefore, the present study aimed to develop an affordable fungal growth medium by using chopped *Portulaca oleracea* plant instead of potato extract in PDB medium. The study further aimed to utilize the extract of an endophytic fungus, assisted by gamma irradiation, for the extracellular synthesis of ZnO NPs. The generated ZnO NPs were then characterized using DLS, XRD, SEM, HRTEM, and UV–Vis. spectroscopy. Finally, the antibacterial and antibiofilm activities of the biosynthesized ZnO NPs were evaluated against selected Gram-positive bacteria.

## Materials and methods

### Chemical

Every chemical and reagent utilized in this investigation was of analytical reagent quality and were purchased from Oxoid and Sigma Aldrich in the United States.

### Gamma radiation

Samples were irradiated using a ^60^Co Gamma Chamber 4000-A (India) in air at a dose rate of 0.895 kGy/h [40, 41].

### Collection of plant samples and isolation of endophytic fungi

To isolate endophytic fungi, fresh *O. basilicum* leaves and stems were gathered from Cairo University's Faculty of Agriculture. The plant materials were thoroughly cleaned under running water, then cut into small pieces and surface-sterilized with 70% ethanol for one minute and 2.5% sodium hypochlorite for two minutes. The segments were rinsed four times with sterile distilled water. The sterilized segments were placed on potato dextrose agar plates (PDA, which comprises 200 g/L of potato, 20 g/L of glucose, and 18 g/L of agar at pH 5.6) supplemented with ampicillin (1 µg/mL) and incubated for 7 days at 27 ± 2 °C after being dried on sterile filter paper under aseptic conditions [42]. Morphological traits, such as colony morphology and reproductive structures, were used to identify fungal isolates. For later usage, purified isolates were kept as slope cultures at 4 °C [43].

### Morphological identification of the endophytic fungi

By cultivating on PDA medium, endophytic fungi were first identified to the species level based on macro- and micro-morphological features such as colony diameter, mycelium color, extracellular exudates, conidial heads, and sporulation. The developed colonies were then analyzed daily based on their morphological features using the universal keys [43].

### Fermentation and extraction of bioactive metabolites from fungal isolates

Potato Dextrose Broth (PDB; 200 g/L potato infusion and 20 g/L glucose at pH 5.6) was used to cultivate the isolates of endophytic fungi from *O. basilicum*. Two 5 mm agar plugs from a 6-day-old culture produced on PDA were used to inoculate each fungal isolate into 250 mL Erlenmeyer flasks with 100 mL of broth medium. The flasks were then incubated at 27 ± 2 °C for 15 days while being shaken at 120 rpm. To get rid of the debris, the culture filtrate was then centrifuged at 4500 rpm after being filtered through sterile cheesecloth. An equivalent volume of dichloromethane (DCM) was added to the cleared filtrate before it was removed using a separating funnel. A rotary evaporator was used to collect and evaporate the organic phase. For upcoming bactericidal tests, the dried residues were then redissolved in Dimethyl Sulfoxide (DMSO) and stored at 4 °C [44].

### Screening of endophytic fungal isolates for antibacterial activity

Using the agar well diffusion method, the antibacterial activity of the extracts of the endophytic fungal isolates was assessed against *B. subtilis* and *S. aureus*. The bacterial inoculum was adjusted to a 0.5 McFarland standard (approximately 1–3 × 10⁸ CFU/mL) using a UV–Vis. spectrophotometer set at 600 nm and uniformly spread over Mueller–Hinton agar plates. Wells (6 mm in diameter) were made aseptically in the agar, and 50 µL of each extract was introduced into the wells. Ciprofloxacin (5 µg) was used as a positive control. Antibacterial efficacy was evaluated by measuring the diameter of the inhibition zones (ZOI) after incubation at 37 °C for 24 h [45].

### Preparation of low-cost fermentation media

In this study we developed two low-cost media. The first was the Potato Peel Medium (PPM) which was prepared by collecting fresh potato peels, thoroughly washing them, and oven-drying them at 40 °C. The dried peels were then ground and reused, and 50 g/l of the dried material was used as a substitute for potato extract in PDB medium. The second medium was *P. oleracea* Medium (POM), which was prepared by collecting the fresh plant, washing it under running water to remove the dust, then chopping it into small pieces. A total of 50 g/l of the chopped plant material was used instead of potato extract in PDB medium. Prior to sterilization, the pH was adjusted to 5.6 prior to autoclaving them for 15 min at 121 °C.

### Evaluation of bioactive metabolite production on different fermentation media

In this experiment, the three most potent fungal isolates were cultivated on three different media: PDB, PPM, and POM, following the fermentation conditions described previously. After extraction, the antibacterial activity of the obtained bioactive metabolites was evaluated using the agar well diffusion method as described in the “Screening of endophytic fungal isolates for antibacterial activity”.

### Molecular identification of the most potent endophyte

Based on the antibacterial activity of bioactive metabolites from the three most potent fungal isolates, the isolate exhibiting the highest inhibition zone was selected for molecular identification. The ITS rDNA region was amplified and sequenced for molecular characterization of the selected isolate [46]. Genomic DNA was extracted from 0.2 g of fungal mycelia after grinding in liquid nitrogen using CTAB buffer (pH 8.0) containing 2% CTAB, 2% PVP40, 0.2% 2-mercaptoethanol, 20 mM EDTA, 1.4 M NaCl, and 100 mM Tris–HCl. PCR amplification of the ITS rDNA region was performed using ITS4 (5′-GAAGTAAAAGTCGTAACAAGG-3′) and ITS5 (5′-TCCTCCGCTTATTGATATGC-3′) primers in a reaction mixture containing i-Taq PCR master mix. The thermal cycling conditions consisted of an initial denaturation at 94 °C for 2 min, followed by 35 cycles of 94 °C for 30 s, 55 °C for 10 s, and 72 °C for 30 s, with a final extension at 72 °C for 2 min. PCR products were analyzed by electrophoresis on a 1.5% agarose gel stained with a 1 kb DNA ladder. The same primer set was used for purification and sequencing of the amplicons. Phylogenetic analysis was conducted using the Maximum Likelihood method based on the Tamura–Nei model with Gamma-distributed rates (5 categories) and 1000 bootstrap replicates, implemented in MEGA version 12 [47].

### Green synthesis of ZnO NPs using fungal bioactive metabolites and gamma-rays

To synthesize ZnO NPs, 5 mL of endophytic fungal extract was combined with an equal volume of 1.0 mM zinc acetate hexahydrate. Before gamma irradiation, the pH of each mixture was brought to neutral (pH 7). The mixture was subsequently subjected to gamma radiation at a dose of 20.0 kGy, in accordance with our earlier investigation [48].

### Characterization of the biosynthesized ZnO NPs

The JASCO V-560 UV–Vis. spectrophotometer was used to measure the optical transmission/absorption spectra of the produced ZnO NPs. The average particle size distribution of the synthesized ZnO NP was determined by dynamic light scattering analysis using a DLS-PSS-NICOMP 380-ZLS particle-size analyzer (St. Barbara, California, USA). An X-ray diffractometer (XRD-6000, Shimadzu Scientific Instruments, Japan) was used to record the ZnO NPs’ X-ray diffraction pattern. The extended X-ray diffraction models were studied using a Cu-Kα target and a nickel filter. XRD patterns were recorded over a 2θ range of 20°–100° at a scanning rate of 2°/min using Cu-Kα radiation (λ = 1.5406 Å) operated at 40 kV and 50 mA. ZnO NPs’ distribution, shape, and border size were examined using a scanning electron microscope (SEM, ZEISS, EVO-MA10, Germany). A high-resolution transmission electron microscope (HR-TEM, JEM2100, Jeol, Japan) was used to examine the biosynthesized ZnO NPs’ form, appearance, and average particle size.

### Antibacterial activity of the biosynthesized ZnO NPs

We tested the antibacterial activity of the synthesized green ZnO NPs as described above in section (Screening of endophytic fungal isolates for antibacterial activity).

### Minimum inhibitory concentration (MIC) determination

Using the serial twofold dilution method, the minimum inhibitory concentration (MIC) was determined in Luria–Bertani (LB) broth. Serial concentrations of the biosynthesized ZnO NPs (1.562–100.0 μg/mL) were prepared in sterile LB medium in 96-well microplates. Each well was inoculated with a standardized bacterial suspension adjusted to 1–3 × 10^8^ CFU/mL prior to dilution. Wells containing bacterial suspension without ZnO NPs served as the growth control (positive control), whereas wells containing sterile LB medium only served as the sterility control (negative control). After incubation at 37 °C for 24 h, bacterial growth was measured spectrophotometrically at 600 nm using a microplate reader. The MIC was defined as the lowest concentration of ZnO NPs that completely inhibited visible bacterial growth [49].

### Antibiofilm activity of the biosynthesized ZnO NPs

As stated by Christensen et al. [49], a semi-qualitative detection of biofilm generation was examined. Both with and without nanoparticles, the visual development of biofilm production at the tube wall was assessed. The antibiofilm activity of biosynthesized ZnO NPs (31.25 µg/mL) against *B. subtilis* and *S. aureus* was evaluated. Three milliliters of nutrient broth were mixed with a modest number (0.5 McFarland (1–3) × 10^8^ CFU/mL) of the bacteria under research, and the mixture was left at 37 °C for a whole day. Following the removal of the generated material, Phosphate Buffer Saline (PBS; pH 7) was added to the treated and control tubes [50]. After drying, the biofilm tubes were stabilized for 10.0 min with 3.0% sodium acetate, and the mixture was rinsed with deionized water. After 15.0 min of staining with 0.1% crystal Violet (CV), the bacterial biofilms were rinsed with deionized water to get rid of any leftover stain. Lastly, the stain was dissolved using 2.0 mL of ethanol. The development of a positive biofilm was demonstrated by a discernible stained layer on the tube's surface and bottom. Bacterial biofilms were estimated to be using a UV–Vis spectrophotometer set at 570 nm, and Eq. (1) was utilized to determine the biofilm inhibition percentage.

1 $$ R\% {\mkern 1mu} = 100{\mkern 1mu} \, \times \,\frac{{\left( {{\mathrm{O}}.{\mathrm{D}}.{\text{ of control sample }} - {\text{ O}}.{\mathrm{D}}.{\text{ of the treated sample}}} \right)}}{{\left( {{\mathrm{O}}.{\mathrm{D}}.{\text{ of control sample}}} \right)}}{\mkern 1mu} {\mkern 1mu} $$

### The ZnO NPs’ impact on bacterial protein permeability

The generated ZnO NPs were combined with 10 mL of the nutrient broth after an 18-h culture of bacteria (*B. subtilis* and *S. aureus*) was adjusted to 1–3 × 10^8^ CFU/mL. Culture-mixed solutions devoid of ZnO NPs were administered to the control group. Following five hours of incubation at 37 °C, the samples were centrifuged for ten minutes at 5000 rpm. One milliliter of Bradford reagent was combined with one hundred microliters of the supernatant extracted from the tested samples. Following a dark incubation of 10 min, optical density was measured at 595 nm [51].

### Statistical analysis

Using Duncan’s multiple range test and the least significant difference (LSD) test, we statistically examined the options at *P* = 0.05 using one-way ANOVA [52]. SPSS software (version 15) was used to analyze and assess the data and findings.

## Results and discussion

### Isolation and morphological identification of endophytic fungi from *Ocimum basilicum*

As shown in Table 1, sixteen endophytic fungal isolates were obtained from different parts of *O. basilicum*, (10 isolates) from the leaves and (6 isolates) from the stem. Using universal keys, fungal isolates were first identified to the species level based on morphological traits [43]. The endophytic fungal isolates were classified to five genera: *Aspergillus*, *Cladosporium*, *Penicillium*, *Fusarium*, and *Alternaria*. *Aspergillus* was the most dominant genus (62.50%) of these isolates, *Penicillium* and *Fusarium* each represented 12.5%, while *Cladosporium* and *Alternaria* were less prevalent, each representing 6.25%. Four species were identified within the genus *Aspergillus*, *A. niger* (4 isolates), *A. flavus* (3 isolates), *A. oryzae* (2 isolates), and *A. tamarii* (1 isolate), as shown in Figure S1.

**Table 1:** Isolation of endophytic fungi from different parts of *Ocimum basilicum*

Plant parts	Isolate code no	Name of fungal isolate
Leaves	1	*A. flavus*_1_
	2	*A. niger*_1_
	3	*Cladosporium* sp.
	4	*Penicillium* sp._1_
	5	*Fusarium* sp._1_
	6	*Alternaria* sp.
	7	*A. tamarii*
	8	*Fusarium* sp._2_
	9	*A. flavus*_2_
	10	*A. niger*_2_
Stem	11	*Penicillium* sp._2_
	12	*A. niger*_3_
	13	*A. oryzae*_1_
	14	*A. flavus*_3_
	15	*A. niger*_4_
	16	*A. oryzae*_2_

### Antibacterial activity of extracted bioactive secondary metabolites from fungal isolates

Out of the sixteen endophytic fungal isolates grown on PDB medium, the fungal bioactive metabolites of *A. tamarii* exhibited the highest antimicrobial activity, followed by *A. flavus*_*3*_ and *A. niger*_*2*_. Moderate antibacterial activity was shown in eight isolates: *A. niger*_1_, *Penicillium* sp._1_, *Fusarium* sp._2_, *A. flavus*_2_, *Penicillium* sp._2_, *A. niger*_3_, *A. oryzae*_1_, and *A. oryzae*_2_. In contrast, five isolates (*A. flavus*_1_, *Cladosporium* sp., *Fusarium* sp._1_, *Alternaria* sp., and *A. niger*_4_) showed no detectable antibacterial activity. The antibacterial potential of the fungal extracts was assessed using the disc diffusion assay against gram-positive bacteria (*B. subtilis* and *S. aureus*). Ciprofloxacin (5 µg; disc diameter 6 mm) was used as positive control. The bioactive metabolites of *A. tamarii* exhibited significant antibacterial activity, generating inhibition zones of 16.0 ± 0.13 mm against *B. subtilis* and 18.0 ± 0.44 mm against *S. aureus*, as indicated by the results displayed in Table 2. According to reports, *A. tamarii* produces bioactive secondary metabolites that have antibacterial action and inhibit both gram-positive bacteria.

**Table 2:** Screening for antibacterial metabolite production by different endophytic fungal isolates of *Ocimum basilicum* on PDB

Isolate code no.	Name of fungal isolates	Antibacterial activity (zone of inhibition; mm ± SD)	
		*B. subtilis*	*S. aureus*
1	*A. flavus*_1_	Nil	Nil
2	*A. niger*_1_	9.0 ± 0.22^h^	15.0 ± 0.12^c^
3	*Cladosporium* sp.	Nil	Nil
4	*Penicillium* sp._1_	10.0 ± 0.23^f^	14.0 ± 0.34^d^
5	*Fusarium* sp._1_	Nil	Nil
6	*Alternaria* sp.	Nil	Nil
7	*A. tamarii*	16.0 ± 0.13^a^	18.0 ± 0.44^a^
8	*Fusarium sp*._2_	13.0 ± 0.33^cd^	12.0 ± 0.32^g^
9	*A. flavus*_2_	9.0 ± 0.44^gh^	14.0 ± 0.22^d^
10	*A. niger*_2_	13.0 ± 0.22^c^	14.0 ± 0.13^de^
11	*Penicillium sp*._2_	14.0 ± 0.23^b^	12.0 ± 0.33^g^
12	*A. niger*_3_	10.0 ± 0.13^fg^	13.0 ± 0.23^f^
13	*A. oryzae*_1_	11.0 ± 0.33^e^	12.0 ± 0.33^g^
14	*A. flavus*_3_	13.0 ± 0.36^cd^	16.0 ± 0.13^b^
15	*A. niger*_4_	Nil	Nil
16	*A. oryzae*_2_	9.0 ± 0.33^h^	11.0 ± 0.12^h^
*LSD*		0.13044	0.12344

*Nil* No ZOI (negative results), *LDS *Least significant differences

The values (n = 3) are means ± SD. One-way analysis of variance (ANOVA) and ^a–h^ Duncan’s multiple range test (DMRT) were used to examine the data within the groups

It was observed that gram-positive bacteria are more sensitive to produced bioactive compounds when compared to gram-negative bacteria [53, 54], that may be attributed to the lack of an outer lipopolysaccharide membrane, allowing easier penetration of lipophilic fungal active metabolites. Several secondary bioactive metabolites have been produced by *A. tamarii* NL3, including griseofulvin, isogriseofulvin, cytochalasin J, solamargine, and solasonine [55]**.** Endophytic *A. tamarii* isolates additionally produce cyclic peptides and indole diketopiperazines with sub-micromolar to low micromolar antimicrobial activity [53]. *A. tamarii* LTRH2 isolated from *Lagenandra toxicaria* demonstrated significant antibacterial activity with inhibition zones of 22.66 ± 0.71 mm against Staphylococcus aureus, 20.00 ± 0.48 mm against *E. faecalis*, and 15.50 ± 0.22 mm against *E. coli* [53]. These results confirm the selectivity for gram-positive bacteria in agar diffusion assays. Broader endophyte screening that included *A. tamarii* also reported measurable halos of approximately 7.0–14.0 mm for ethyl acetate extracts, again favoring gram-positive targets [54].

### Evaluation of cost-effective fermentation media in comparison with PDB for bioactive compound production

The obtained results showed that *A. tamarii* cultivated on POM medium produced higher levels of bioactive metabolites with inhibition zones of 20.0 ± 0.33 mm against *B. subtilis* and 23.0 ± 0.26 mm against *S. aureus*, this was followed by PPM and PDB media. The obtained results can be attributed to the phytochemical composition of *P. oleracea* substrate, which is rich in compounds (phenolics, flavonoids, carotenoids, omega-3 fatty acids and organic acids), that are known to modulate fungal metabolic pathways and stimulate the biosynthesis of bioactive secondary metabolites, particularly under nutrient-complex conditions [56, 57].

In contrast, potato-based media provide mainly starch and simple nutrients, supporting good fungal growth but offering limited chemical signals that induce metabolite diversification. Potato peel medium, although economical, still lacks the phytochemical complexity required to trigger strong metabolite induction. The superior performance of the purslane substrate therefore suggests that plant-derived bioactive compounds acted as metabolic elicitors, leading to increased production of antioxidant, antimicrobial, or reducing metabolites that enhance biological activity or nanoparticle synthesis [58]. As we know, there is no published study that has systematically compared *A. tamarii* cultivation on *P. oleracea* substrate with conventional potato media or evaluated whether purslane-derived phytochemicals serve as metabolic elicitors for this fungal species (Table 3).

**Table 3:** Antibacterial metabolite production by different endophytic fungal isolates of *Ocimum basilicum* on different media

Microbes	*A. tamarii*			*A. niger*_*2*_			*A. flavus*_*3*_			CIP (PO media)
	PDB	PPM	PO	PDB	PP	PO	PDB	PP	PO	
	Antibacterial activity (zone of inhibition; mm ± SD)									
*B. subtilis*	16.0 ± 0.13^f^	17.0 ± 0.16^e^	20.0 ± 0.33^b^	13.0 ± 0.45^de^	14.0 ± 0.18^ cd^	19.0 ± 0.23^b^	13.0 ± 0.22^e^	15.0 ± 0.18^c^	14.0 ± 0.23^de^	35.0 ± 0.23^a^
*S. aureus*	18.0 ± 0.23^d^	20.0 ± 0.22^c^	23.0 ± 0.26^a^	14.0 ± 0.24^c^	12.0 ± 0.40^e^	21.0 ± 0.13^a^	16.0 ± 0.30^bc^	16.0 ± 0.18^b^	18.0 ± 0.55^a^	32.0 ± 0.45^b^
*LSD*	0.12233			0.13000			0.17383			0.20222

*PDB* Potato Dextrose Broth, *PPL* Potato Peel, *PO* Portulaca oleracea, *CIP* Ciprofloxacin (Standard antibacterial agent)

The values (n = 3) are means ± SD. One-way analysis of variance (ANOVA) and ^a–f^ Duncan's multiple range test (DMRT) were used to examine the data within the groups. LDS = Least significant differences

### Molecular identification of the most potent endophytic fungi

**Fig. 1:**
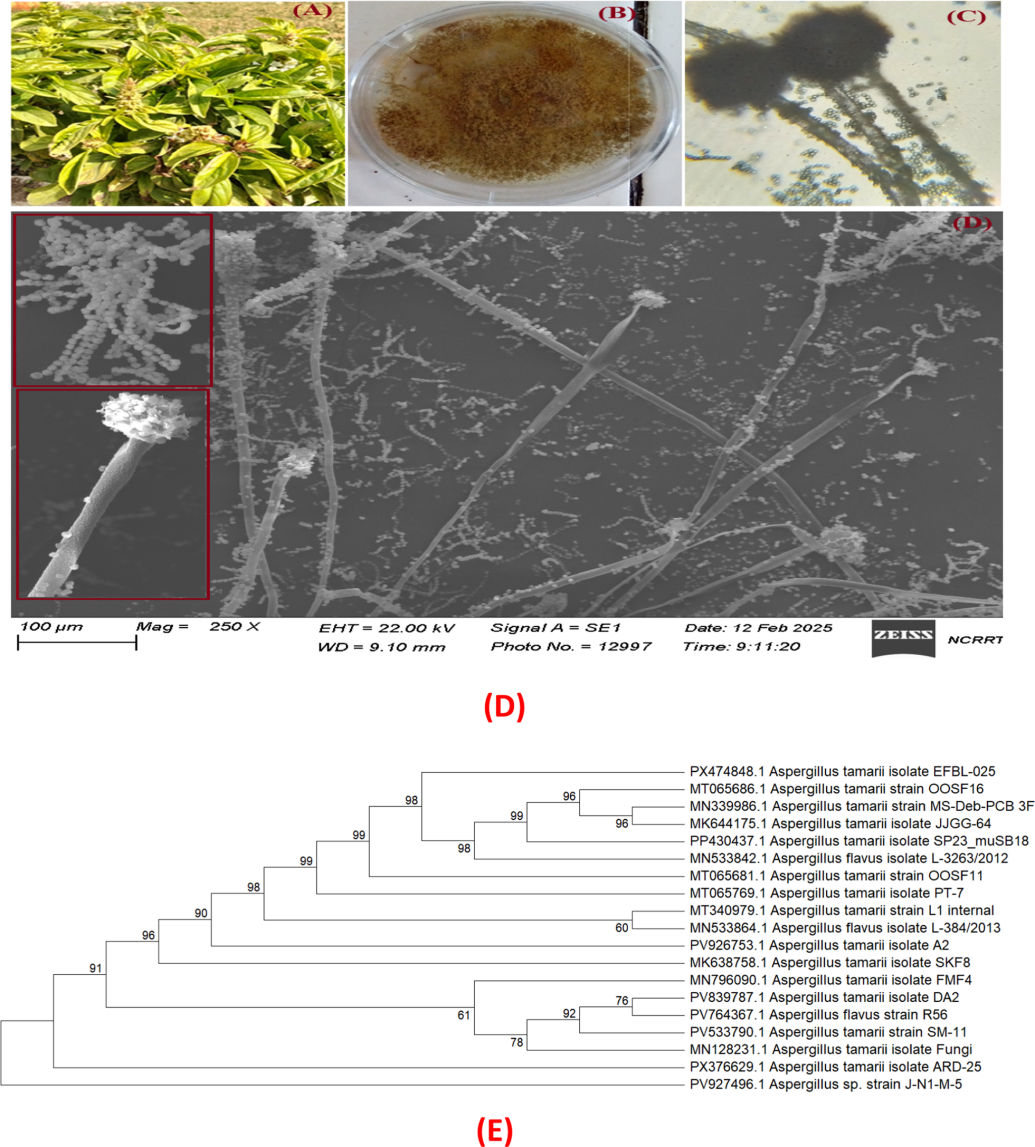
**A** Morphological views of *Ocimum basilicum* plant, **B** The plate culture and conidial heads of *A. tamarii* an endophyte of *Ocimum basilicum,* **C** Microscopical views of isolated conidial heads, **D** SEM for *A. tamarii* conidiophore, conidial heads and spores, and **E** Phylogenetic analysis of ITS *A. tamarii* by maximum likelihood method

The resulting ITS sequence was subjected to Nucleotide BLAST (BLASTn) analysis against the NCBI database, confirming its identity as *A. tamarii* with 100% sequence identity and 100% query coverage (E-value = 0.0) to multiple reference strains (e.g., MT065686.1 and MN339986.1), The isolate clustered together with authenticated *A. tamarii* reference sequences with strong bootstrap support (≥99%) at key nodes. The sequence was deposited in GenBank under accession number PX474848.1 (Fig. 1).

### Effect of gamma rays on ZnO NPs synthesis

**Fig. 2:**
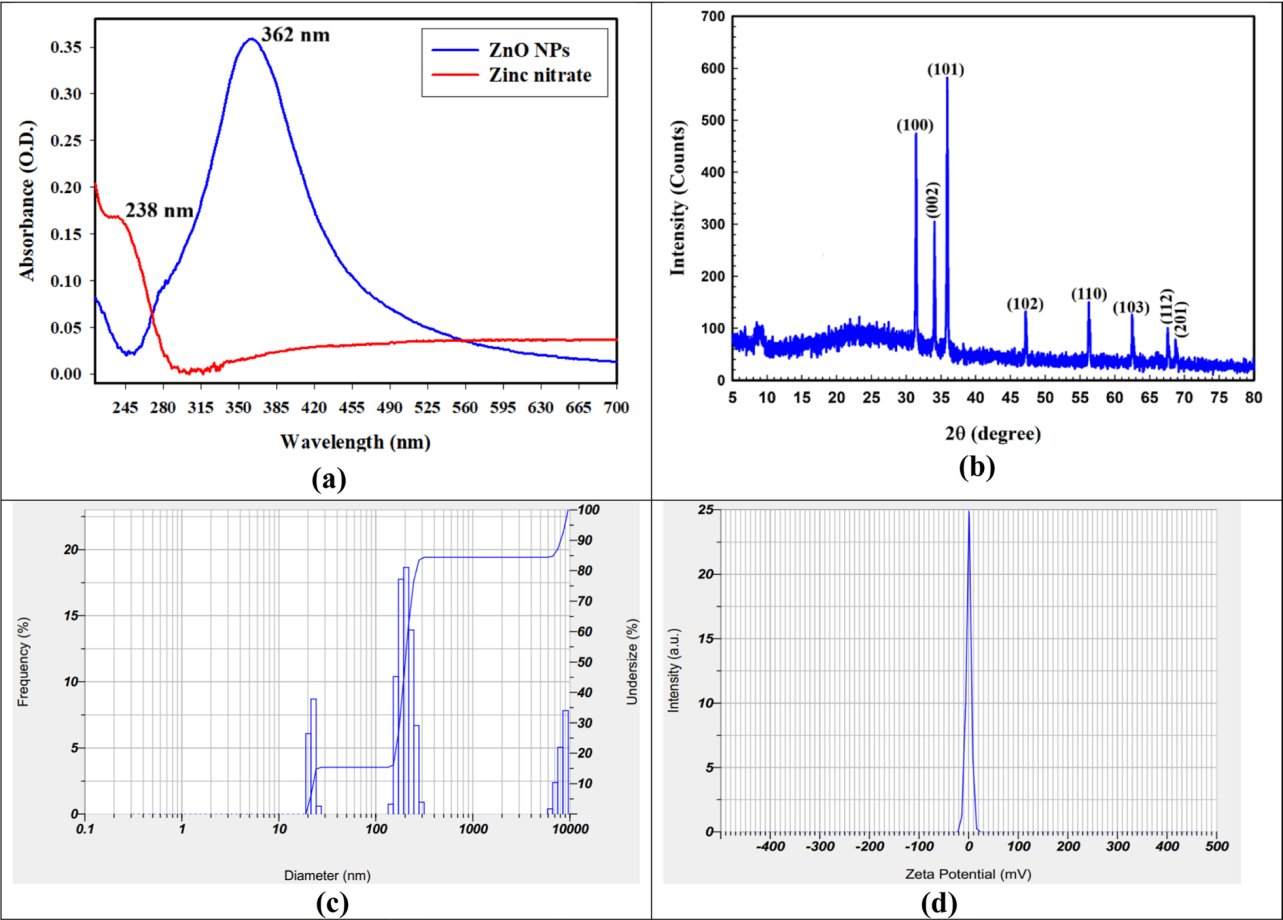
**a** UV–Vis. spectrophotometry, **b** XRD analysis, **c** DLS determination, and **d** zeta potential for the synthesis of ZnO NPs using gamma-rays and fungal filtrate

Figure 2a illustrates the process of creating ZnO NPs using gamma rays, based on UV–Vis. spectrophotometry. As shown in Fig. 2a, 20 kGy was used for producing ZnO NPs, as shown by the O.D. of 0.36 (diluted three times) at 362.0 nm and the zinc nitrate solution was detected at 238.0 nm with O.D. of 0.17. Gamma rays made it possible to make uniform ZnO NPs with a high relative yield [59].

This approach eliminates the need for high temperatures and other chemicals that break things down. The production of ZnO NPs is not aided by excessive radiation because solvated electrons and excess free radicals generated by water radiolysis can alter the pH of solutions, attack the oppositely charged ZnO NPs, interact with them, and eventually form aggregated ZnO NPs that lose intensity in the UV–Vis spectrum [60]. A review of the literature [61, 62] noted that metal-based nanoparticles are easy to make, kill bacteria, and can be used in many situations, like keeping food safe from certain invasive pathogenic microorganisms. They were also applied to tissue engineering.

### Characterization of the synthesized ZnO NPs

#### XRD, DLS, and Zeta potential determination

The XRD spectrum of ZnO NPs exhibits unique diffraction patterns at certain 2θ angles between 5° and 80° (Fig. 2b). The XRD peaks at 31.4°, 34.05°, 35.88°, 47.17°, 56.23°, 62.50°, 67.59°, and 68.73°, correspond to the (100), (002), (101), (102), (110), (103), (112), and (201) reflections, respectively. The acquired data were compared with the JCPDS database for ZnO nanoparticles (card no. #01–1136), which corresponds to the hexagonal structure of ZnO NPs [63].

Finally, when looking at the fungal filtrate (Fig. 2b), there is only one weak peak at 22.85° that is related to the stabilization of the synthesized ZnO NPs. The XRD data showed that the ZnO NPs that were made were very crystal-like, and they joined with an amorphous fungal extract, which made it easier for them to spread out in the solution and make it work better [64].

Lastly, the Williamson-Hall (W H) equation [65, 66], determined the intermediate ZnO NPs crystallite size, which was found to be 20.20 nm in accordance with Eq. (2).

2 $$\beta \mathrm{cos}\theta =\frac{k\lambda }{{D}_{W-H}}+4\varepsilon \mathrm{sin}\theta $$

According to the DLS approach, the synthesized ZnO NPs, which were created using gamma rays and fungal filtrate, had an average particle size distribution of 21.9 and 204.2 nm (Fig. 2c). Samples are considered monodisperse by the International Standards Organization (ISO) if the polydispersity index (PDI) findings are less than 0.05. On the other hand, particles with a polydispersity distribution are expected to be produced when PDI results are higher than 0.7 [67]. Our data showed that the generated ZnO NPs had PDI values of 0.95. The ZnO NPs that were produced represented a respectable range of polymers based on the current values. Because DLS is scientifically valid, the nanoparticles that are made are spread out over a narrow or uniform size. This improves their properties and biological uses [68].

The average and primary sizes determined by DLS analysis were larger than the particle sizes estimated by HRTEM imaging, according to the results. The radius of hydrodynamics inside the created ZnO NPs and the water-based layers surrounding them are responsible for the significant sizes of the synthesized bimetallic NPs, according to the literature review [69].

The zeta potential of the generated ZnO NPs was determined at a pH of 7.2, as seen in Fig. 2d. The present results show that at the pH of the investigated solution, the naturally occurring ZnO NPs retain a negative surface zeta potential. Furthermore, as illustrated in Fig. 2d, the preparation's zeta potential at a neutral medium (pH 7.2) was −0.1 mV due to the negative electrical charge of the fungal filtrate component.

#### HRTEM and SEM imaging

HRTEM imaging was used to find the average particle size and show how the ZnO NPs that were made looked. Additionally, DLS measurements and HRTEM data were contrasted.

**Fig. 3:**
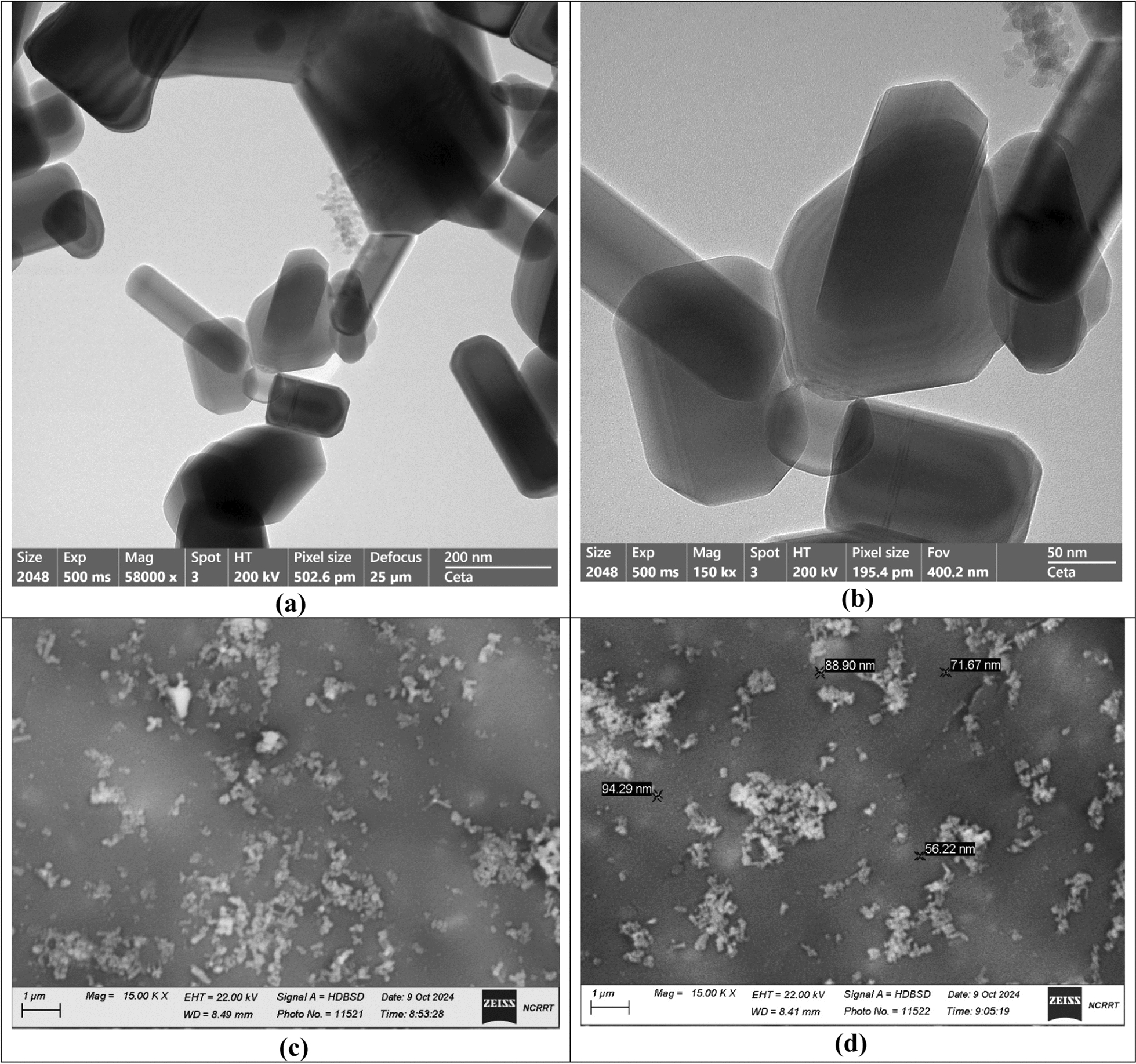
Average particle size, shape, and surface morphology for the synthesized ZnO NPs where **a**, **b** HRTEM imaging, and **c**, **d** SEM imaging

As you can see in Fig. 3a, the ZnO NPs that were made had different shapes, such as rod and half-spherical shapes, which were shown by HRTEM images. The scale of ZnO NPs, which had an average diameter of 70.34 nm and ranged from 30.22 to 90.23 nm, is depicted in Fig. 3b. A comparison of the average particle size and shape in the literature revealed that the synthesized ZnO NPs (in this work) were small and primarily spherical and rod-shaped. The manufactured shapes may differ in the literature review since extracted NPs were nearly spherical or ellipsoidal in all cases [70]. However, because of the synthetic process from the extract, various morphologies may be detected; therefore, the anisotropic shape was recorded. Because we used more than one reducing agent (gamma rays and parts of fungal filtrates), our study showed a different shape (anisotropic structure).

Figure 3c, d displays the surface morphology of the generated ZnO NPs. Bright ZnO NPs were continuously present in pure condition, as seen in the image in Fig. 3c, d. The ZnO nanoparticles made in this study were evenly spread out, small, and had the same spherical shape. We discovered this by comparing their morphological shape and elemental analysis to those from previous research.

Mohsin et al. [71] used the citrate reduction process to produce metallic silver and gold nanoparticles at different pH levels and temperatures. Since the observed morphological shape and border size showed that they ranged from 50 to 65 nm and appeared to be spherical particles, temperature and pH are important elements in the creation process. EDX examined the elemental structure of ZnO NPs and contrasted it with the most recent publication [71], showing that ZnO NPs were produced in pure form. Lastly, our findings were connected to the previously released papers [72–75].

#### Antibacterial activity of the biosynthesized ZnO NPs

The antibacterial activity of ZnO NPs biosynthesized using fungal extracts of *A. tamarii* cultivated on PDB, PPM, and POM media was evaluated against *B. subtilis* and *S. aureus*. As presented in Table 4, ZnO NPs obtained from the POM-derived extract exhibited the highest antibacterial activity, producing inhibition zones of 26.0 ± 0.30 and 27.0 ± 0.13 mm against *B. subtilis* and *S. aureus*, respectively, with identical MIC values of 31.25 µg/mL for both strains.

**Table 4:** Antibacterial activity of synthesized ZnO NPs as ZOI (mm), and MIC (µg/mL)

Microbes	*A. tamarii*						CIP (PO media)
	PDB	PPM	PO	PDB	PP	PO	
	(ZOI; mm ± SD)			(MIC; µg/mL)			(ZOI; mm ± SD)
*B. subtilis*	19.0 ± 0.18^ cd^	17.0 ± 0.14^f^	26.0 ± 0.30^b^	62.50	125.0	31.25	35.0 ± 0.23^a^
*S. aureus*	18.0 ± 0.31^de^	19.0 ± 0.20^c^	27.0 ± 0.13^a^	62.50	62.50	31.25	32.0 ± 0.45^b^
*LSD*	0.11344			–			0.20222

*PDB* Potato Dextrose Broth, *PPL* Potato Peel, *PO* *Portulaca oleracea*, *CIP* Ciprofloxacin (Standard antibacterial agent)

The values (n = 3) are means ± SD. One-way analysis of variance (ANOVA) and ^a–f^ Duncan's multiple range test (DMRT) were used to examine the data within the groups. LDS = Least significant differences

In comparison, ZnO NPs synthesized from PDB-derived extracts showed moderate activity, with inhibition zones of 19.0 ± 0.18 mm and 18.0 ± 0.31 mm against *B. subtilis* and *S. aureus*, respectively, and MIC values of 62.5 µg/mL. The lowest antibacterial effect was observed for ZnO NPs synthesized from PPM-derived extracts, particularly against *B. subtilis* (17.0 ± 0.14 mm; MIC = 125 µg/mL), while activity against *S. aureus* reached 19.0 ± 0.20 mm (MIC = 62.5 µg/mL). The obtained results demonstrate the biosynthesized ZnO NPs' strong antibacterial activity, which is probably due to their unique physicochemical properties and nanoscale size. Even at low concentrations, the ZnO NPs showed strong inhibitory effects, indicating their potential as effective antimicrobial agents.

Among the characteristics of inorganic metal oxide nanoparticles are their small size and high surface-to-volume ratio. They can display distinctive and important activities when dealing with some infectious pathogens, such as bacteria. The electrostatic interaction that ultimately results in cell membrane damage, disrupts proteins and enzymes, produces reactive oxygen species (ROS) and oxidative stress, prevents proteins from binding to homeostasis (electronic transport chain disruption), halts signal transduction, and damages genes may be the cause of ZnO NPs' antibacterial activity [76, 77].

The roughness of the ZnO NPs' exterior surface may also be responsible for their efficacy; this damage to the cell wall allows the ZnO NPs to reach the plasma membrane, where they kill bacteria [78]. Because of their unique properties, inorganic nanoparticles can be applied in a wide range of medical applications. Moreover, by increasing the resistance of some bacteria to traditional antibiotics, they reduce their effectiveness and treatment potential [79]. ZnO NPs maintained advantageous physicochemical characteristics, such as special interaction mechanisms, that improved their efficacy against more resistant bacteria and yeast, increasing their total antibacterial activity in comparison to the majority of manufactured antimicrobials [80].

Nevertheless, it is unclear how ZnO NPs work as an antimicrobial. Complex techniques were used to disperse reactive oxygen species (ROS), also known as superoxide anion (O_2_^−^) [81]. The antibacterial action was demonstrated by the pathogenic bacteria's combination of nano-silica and an alkaline inclination [82]. According to some theories, ZnO NPs may alter the ability of germs to cross membranes, the formation of microbe films, and the way oxidative stress genes react to the generation of H_2_O_2_ [83].

#### Antibiofilm activity the biosynthesized ZnO NPs

**Fig. 4:**
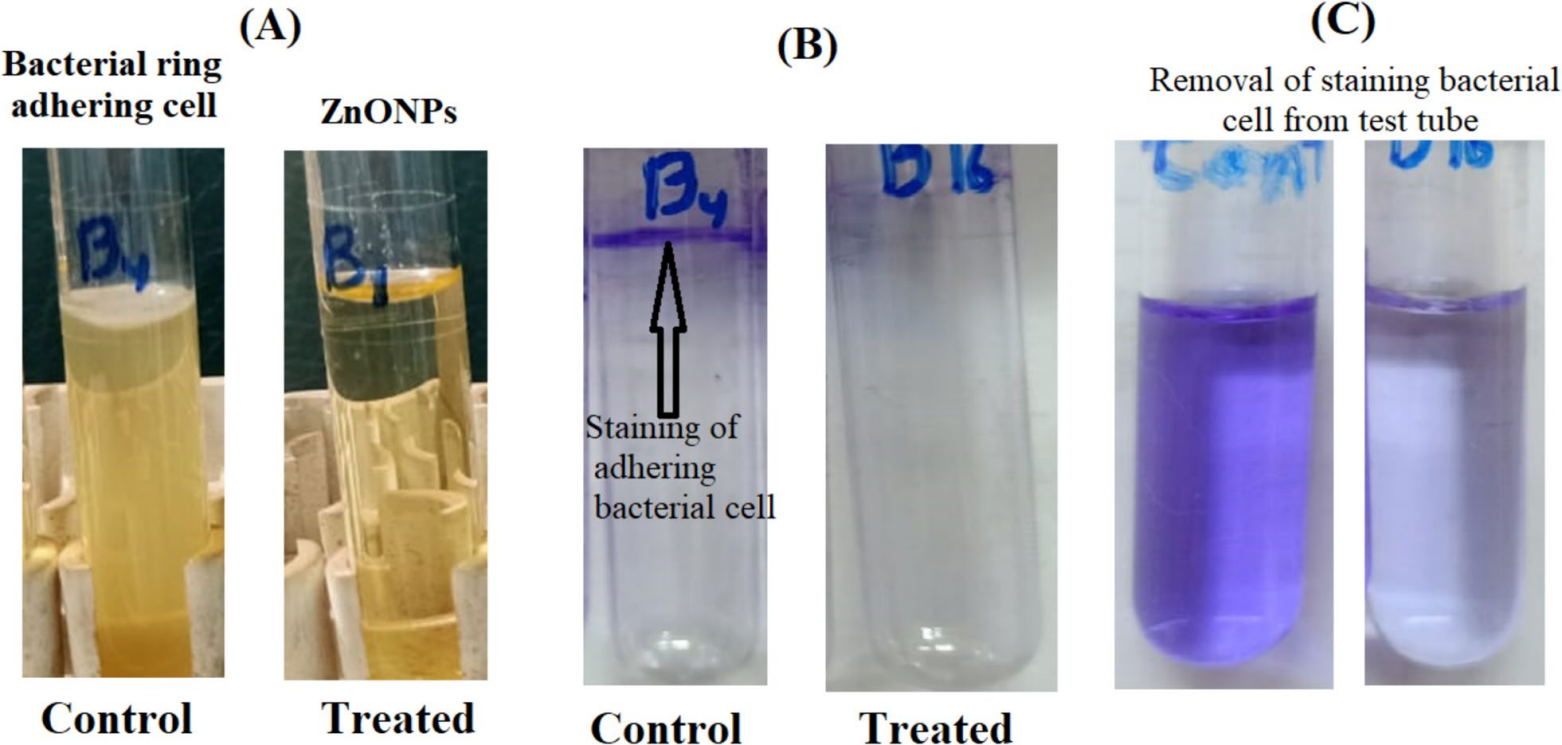
Antibiofilm activity of ZnO NPs against *B. subtilis* where **A** Test method for biofilm detection in the presence and absence of ZnO NPs, **B** Staining of the adherent bacterial cell using Crystal Violet stain, and **C** Crystal Violet decolorization by ethanol for quantitative analysis

The tube method was used to assess *B. subtilis* and *S. aureus* biofilm development. Untreated control tubes displayed a dense, yellowish-yellow biofilm mat at the air–liquid interface following a 24-h incubation period at 37 °C (Fig. 4A).

On the other hand, ZnO NP-treated tubes displayed a weak white-yellow biofilm mat at the air–liquid interface, as indicated by reduced adhering material, disturbed ring formation, and mild staining. The biofilm showed up as a deep violet layer when stained with crystal violet (CV). Semi-quantitative analysis was performed using the intensity of the violet solution that resulted from dissolving the CV in ethanol (Fig. 4B).

The percentage of bacterial biofilm formation inhibition was measured using a UV–Vis. spectrophotometer set to 570.0 nm. The O.D. was measured following the dissolution of the pigmented biofilm in ethanol. According to Table 5, the percentage of inhibition against *B. subtilis* and *S. aureus* was 50.32 and 55.33%, respectively.

**Table 5:** Antibiofilm activity of synthesized ZnO NPs as biofilm inhibition percentage

Microbes	*A. tamarii*		
	Control	PO	Biofilm inhibition %
	O.D of C.V stain @ 570.0 nm		
*B. subtilis*	0.759 ± 0.034^a^	0.382 ± 0.041^c^	50.32
*S. aureus*	0.515 ± 0.031^b^	0.285 ± 0.018^d^	55.33
*LSD*	0.01022		–

*PO* *Portulaca oleracea*

The values (n = 3) are means ± SD. One-way analysis of variance (ANOVA) and ^a–d^ Duncan's multiple range test (DMRT) were used to examine the data within the groups. LDS = Least significant differences

NPs can continue to affect the biofilm produced in the second step, causing it to spread by penetrating the film and eliminating the microbial cells. Consequently, we think that NP-based antibiofilm coatings may be used as probes for biofilm removal, imaging, and therapy. Their minimal toxicity and wide spectrum of activity are further advantages. As a result, employing semiconductor nanoparticles may offer both visual observation and inhibitory process detection [84].

### Bacterial protein leakage investigation

**Fig. 5:**
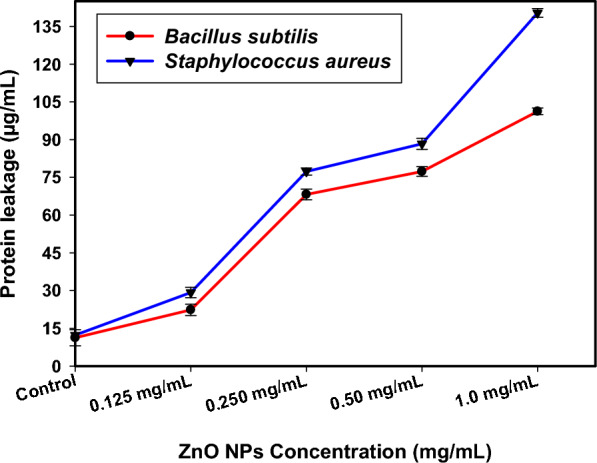
The effect of green synthesized ZnO NPs on the protein leakage from *S. aureus*, and *B. subilits* cell membranes

The Bradford method was used to calculate the protein outflow quantities in the treated solutions of *S. aureus* and *B. subtilis* [85]. Figure 5 shows a direct correlation between the amount of S. aureus protein removed and the amount of generated ZnO NPs (at varying doses). After being incubated with ZnO NPs (1.0 mg/mL), the count was 101.23 µg/mL.

Furthermore, with counts after ZnO NPs implementation being 140.40 µg/mL at the specified ZnO NPs concentration (1.0 mg/mL), Fig. 5 shows how the concentration of green ZnO NPs exactly correlates with the amount of *B. subtilis* protein eliminated. This illustrates how holes grow in the membranes of *B. subtilis* and *S. aureus*, which aid in the proteins' escape from the bacterial cytoplasm and demonstrate the antibacterial properties of green-synthesized ZnO NPs.

The findings demonstrated that ZnO NPs enhanced the breakdown of *B. subtilis* and *S. aureus* membranes and altered their permeability. The metal's positive impact on bacterial membrane permeability, which eventually results in protein leakage, is the main factor preventing bacterial development.

Comparable investigations, such as [86, 87], provide similar results after NP treatment, revealing bacterial internal organelles seeping through the exterior cell structure and indicating concentration dependence for bacterial membrane dislodgement.

Paul et al., [88], assert that the rate of dissimilarity in connected electric conductivity drives the bacteria's dissimilarity in membrane permeability. The protein leakage test is one of the most crucial techniques for assessing the skeletal strength of any microbe. Eventually, leakage developed into regular microbial damage, and cell collapse resulted from the release of cell components.

## Conclusion

This work presents an economical technique for the synthesis of bioactive metabolites from endophytic fungi as well as a green and economical method for the synthesis of ZnO NPs utilizing gamma radiation induction and fungal extract. With a distinct zone of inhibition of about 20.0 mm against *B. subtilis* and 23.0 mm against *S. aureus*, the fungal extract of *A. tamarii* grown on POM medium demonstrated strong antibacterial activity. Next, fungal extract and gamma radiation were used to create ZnO NPs. The resultant nanoparticles had an average diameter of 70.34 nm and were primarily spherical and rod-shaped. According to the suggested reaction mechanism, gamma irradiation at 20.0 kGy might improve metal ion reduction by interacting oxidatively with fungal metabolites. UV–Vis. spectroscopy, DLS, XRD, SEM, and SEM were used to characterize and validate the biosynthesized ZnO NPs. ZnO NPs have an average diameter of 70.34 nm with a spherical and rod-like form. With minimum inhibitory concentrations (MICs) of 31.25 µg/ml, respectively, the produced ZnO NPs showed strong antibacterial activity against *S. aureus* (27.0 mm; ZOI) and *B. subtilis* (26.0 mm; ZOI). This study presents the economical and environmentally friendly biosynthesis of ZnO NPs, a promising antibacterial agent against Gram-positive bacteria.

## Supplementary Information

Additional file 1.

## Data Availability

The data that support the findings of this study are available from corresponding authors, but restrictions apply to the availability of these data, which were used under license for the current study, and so are not publicly available. Data are however available from the authors upon reasonable request and with permission of corresponding authors.

## References

[1] Chinemerem Nwobodo D, Ugwu MC, Oliseloke Anie C, Al-Ouqaili MT, Chinedu Ikem J, Victor Chigozie U, et al. Antibiotic resistance: the challenges and some emerging strategies for tackling a global menace. J Clin Lab Anal. 2022;36:e24655. 35949048 10.1002/jcla.24655 PMC9459344

[2] Salam MA, Al-Amin MY, Salam MT, Pawar JS, Akhter N, Rabaan AA, Alqumber MA: Antimicrobial resistance: a growing serious threat for global public health. In *Healthcare*. Basel: Multidisciplinary Digital Publishing Institute; 2023. 10.3390/healthcare11131946 PMC10340576 37444780

[3] Mubeen B, Ansar AN, Rasool R, Ullah I, Imam SS, Alshehri S, et al. Nanotechnology as a novel approach in combating microbes providing an alternative to antibiotics. Antibiotics. 2021;10:1473. 34943685 10.3390/antibiotics10121473 PMC8698349

[4] Nair GM, Sajini T, Mathew B. Advanced green approaches for metal and metal oxide nanoparticles synthesis and their environmental applications. Talanta Open. 2022;5:100080.

[5] Mendes CR, Dilarri G, Forsan CF, Sapata VdMR, Lopes PRM, de Moraes PB, et al. Antibacterial action and target mechanisms of zinc oxide nanoparticles against bacterial pathogens. Sci Rep. 2022;12:2658. 35173244 10.1038/s41598-022-06657-y PMC8850488

[6] Scott LN, Fiume M, Zhu J, Bergfeld WF, Belsito DV, Hill RA, et al. Safety assessment of zinc salts as used in cosmetics. Int J Toxicol. 2024;43:5S-69S. 38279815 10.1177/10915818241227124

[7] Islam F, Shohag S, Uddin MJ, Islam MR, Nafady MH, Akter A, et al. Exploring the journey of zinc oxide nanoparticles (ZnO-NPs) toward biomedical applications. Materials. 2022;15:2160. 35329610 10.3390/ma15062160 PMC8951444

[8] Kim I, Viswanathan K, Kasi G, Thanakkasaranee S, Sadeghi K, Seo J. ZnO nanostructures in active antibacterial food packaging: preparation methods, antimicrobial mechanisms, safety issues, future prospects, and challenges. Food Rev Int. 2022;38:537–65.

[9] Subramaniam VD, Prasad SV, Banerjee A, Gopinath M, Murugesan R, Marotta F, et al. Health hazards of nanoparticles: understanding the toxicity mechanism of nanosized ZnO in cosmetic products. Drug Chem Toxicol. 2019;42:84–93. 30103634 10.1080/01480545.2018.1491987

[10] Ong CB, Ng LY, Mohammad AW. A review of ZnO nanoparticles as solar photocatalysts: synthesis, mechanisms and applications. Renew Sustain Energy Rev. 2018;81:536–51.

[11] Sabir S, Arshad M, Chaudhari SK. Zinc oxide nanoparticles for revolutionizing agriculture: synthesis and applications. Sci World J. 2014;2014:925494. 10.1155/2014/925494 PMC4243478 25436235

[12] Noman MT, Amor N, Petru M. Synthesis and applications of ZnO nanostructures (ZONSs): a review. Crit Rev Solid State Mater Sci. 2022;47:99–141.

[13] Uribe-López M, Hidalgo-López M, López-González R, Frías-Márquez D, Núñez-Nogueira G, Hernández-Castillo D, et al. Photocatalytic activity of ZnO nanoparticles and the role of the synthesis method on their physical and chemical properties. J Photochem Photobiol A Chem. 2021;404:112866.

[14] Kalpana V, Devi Rajeswari V. A review on green synthesis, biomedical applications, and toxicity studies of ZnO NPs. Bioinorg Chem Appl. 2018;2018:3569758. 30154832 10.1155/2018/3569758 PMC6093006

[15] Ameen F, Dawoud T, AlNadhari S. Ecofriendly and low-cost synthesis of ZnO nanoparticles from *Acremonium potronii* for the photocatalytic degradation of azo dyes. Environ Res. 2021;202:111700. 34274331 10.1016/j.envres.2021.111700

[16] Chong WJ, Shen S, Li Y, Trinchi A, Simunec DP, Kyratzis IL, et al. Biodegradable PLA-ZnO nanocomposite biomaterials with antibacterial properties, tissue engineering viability, and enhanced biocompatibility. Smart Mater Manuf. 2023;1:100004.

[17] Szczyglewska P, Feliczak-Guzik A, Nowak I. Nanotechnology–general aspects: a chemical reduction approach to the synthesis of nanoparticles. Molecules. 2023;28:4932. 37446593 10.3390/molecules28134932 PMC10343226

[18] Egbuna C, Parmar VK, Jeevanandam J, Ezzat SM, Patrick-Iwuanyanwu KC, Adetunji CO, et al. Toxicity of nanoparticles in biomedical application: nanotoxicology. J Toxicol. 2021;2021:9954443. 34422042 10.1155/2021/9954443 PMC8376461

[19] Nie P, Zhao Y, Xu H. Synthesis, applications, toxicity and toxicity mechanisms of silver nanoparticles: a review. Ecotoxicol Environ Saf. 2023;253:114636. 36806822 10.1016/j.ecoenv.2023.114636

[20] Rahman A, Harunsani MH, Tan AL, Khan MM. Zinc oxide and zinc oxide-based nanostructures: biogenic and phytogenic synthesis, properties and applications. Bioprocess Biosyst Eng. 2021;44:1333–72. 33661388 10.1007/s00449-021-02530-w

[21] Koza NA, Adedayo AA, Babalola OO, Kappo AP. Microorganisms in plant growth and development: roles in abiotic stress tolerance and secondary metabolites secretion. Microorganisms. 2022;10:1528. 36013946 10.3390/microorganisms10081528 PMC9415082

[22] Sportelli MC, Gaudiuso C, Volpe A, Izzi M, Picca RA, Ancona A, et al. Biogenic synthesis of ZnO nanoparticles and their application as bioactive agents: a critical overview. Reactions. 2022;3:423–41.

[23] Hammad SE, El-Rouby MN, Abdel-Aziz MM, El-Sayyad GS, Elshikh HH. Endophytic fungi–assisted biomass synthesis of gold, and zinc oxide nanoparticles for increasing antibacterial, and anticancer activities. Biomass Convers Biorefin. 2025;15:2285–302.

[24] Pandit C, Roy A, Ghotekar S, Khusro A, Islam MN, Emran TB, et al. Biological agents for synthesis of nanoparticles and their applications. J King Saud Univ Sci. 2022;34:101869.

[25] Brar KK, Magdouli S, Othmani A, Ghanei J, Narisetty V, Sindhu R, et al. Green route for recycling of low-cost waste resources for the biosynthesis of nanoparticles (NPs) and nanomaterials (NMs)-a review. Environ Res. 2022;207:112202. 34655607 10.1016/j.envres.2021.112202

[26] Agrawal K, Gupta VK, Verma P. Microbial cell factories a new dimension in bio-nanotechnology: exploring the robustness of nature. Crit Rev Microbiol. 2022;48:397–427. 34555291 10.1080/1040841X.2021.1977779

[27] Sudheer S, Bai RG, Muthoosamy K, Tuvikene R, Gupta VK, Manickam S. Biosustainable production of nanoparticles via mycogenesis for biotechnological applications: a critical review. Environ Res. 2022;204:111963. 34450157 10.1016/j.envres.2021.111963

[28] Adeleke BS, Olowe OM, Ayilara MS, Fasusi OA, Omotayo OP, Fadiji AE, et al. Biosynthesis of nanoparticles using microorganisms: a focus on endophytic fungi. Heliyon. 2024. 10.1016/j.heliyon.2024.e39636. 39553612 10.1016/j.heliyon.2024.e39636 PMC11564013

[29] Verma N, Jujjavarapu SE, Mahapatra C. Green sustainable biocomposites: substitute to plastics with innovative fungal mycelium based biomaterial. J Environ Chem Eng. 2023;11:110396.

[30] Fathy RM, Mahfouz AY. Eco-friendly graphene oxide-based magnesium oxide nanocomposite synthesis using fungal fermented by-products and gamma rays for outstanding antimicrobial, antioxidant, and anticancer activities. J Nanostruct Chem. 2021;11:301–21.

[31] El-Sayed E-SR, El-Sayyad GS, Abdel-Fatah SS, El-Batal AI, Boratyński F. Novel nanoconjugates of metal oxides and natural red pigment from the endophyte *Monascus ruber* using solid-state fermentation. Microb Cell Fact. 2024;23:259. 39343880 10.1186/s12934-024-02533-8 PMC11439306

[32] Amin RM, Mahmoud RK, Gadelhak Y, El-Ela FIA. Gamma irradiated green synthesized zero valent iron nanoparticles as promising antibacterial agents and heavy metal nano-adsorbents. Environ Nanotechnol Monit Manag. 2021;16:100461.

[33] Zikalala NE, Azizi S, Thema FT, Cloete KJ, Zinatizadeh AA, Mokrani T, et al. Modification of graphene-based nanomaterials with gamma irradiation as an eco-friendly approach for diverse applications: a review. FlatChem. 2024. 10.1016/j.flatc.2024.100662.

[34] Proctor CA. Biology and control of common purslane (*Portulaca oleracea* L.). Lincoln: The University of Nebraska-Lincoln; 2013. p. 2013.

[35] Alam AM, Juraimi AS, Rafii M, Hamid AA, Uddin KM, Alam M, et al. Genetic improvement of purslane (*Portulaca oleracea* L.) and its future prospects. Mol Biol Rep. 2014;41:7395–411. 25085039 10.1007/s11033-014-3628-1

[36] Shanker N. Sustainable extraction and utilization of underulised plant purslane (Portulaca Oleracea). In: Food Product Formulations in Sustainable Food Systems (Volume II) SFS: Novel Sustainable Green Technologies, Circular Strategies, Food Safety & Diversity. p. 69–76. Cham: Springer; 2023.

[37] Naeem F, Khan SH. Purslane (*Portulaca oleracea* L.) as phytogenic substance—a review. J Herbs Spices Med Plants. 2013;19:216–32.

[38] Vescovo D, Manetti C, Ruggieri R, Spizzirri UG, Aiello F, Martuscelli M, et al. The valorization of potato peels as a functional ingredient in the food industry: a comprehensive review. Foods. 2025;14:1333. 40282735 10.3390/foods14081333 PMC12026436

[39] Sawicka B, Khan J. Food and agricultural byproducts as important source of valuable nutraceuticals. Cham: Springer; 2022.

[40] Sivaselvam S, Selvakumar R, Viswanathan C, Ponpandian N. Rapid one-pot synthesis of PAM-GO-Ag nanocomposite hydrogel by gamma-ray irradiation for remediation of environment pollutants and pathogen inactivation. Chemosphere. 2021;275:130061. 33677277 10.1016/j.chemosphere.2021.130061

[41] El-Batal AI, Nada HG, El-Behery RR, Gobara M, El-Sayyad GS. Nystatin-mediated bismuth oxide nano-drug synthesis using gamma rays for increasing the antimicrobial and antibiofilm activities against some pathogenic bacteria and Candida species. RSC Adv. 2020;10:9274–89. 35497243 10.1039/c9ra10765g PMC9050052

[42] Abdel-Fatah SS, El-Batal AI, El-Sherbiny GM, Khalaf MA, El-Sayed AS. Production, bioprocess optimization and γ-irradiation of *Penicillium polonicum*, as a new Taxol producing endophyte from *Ginko biloba*. Biotechnol Rep. 2021;30:e00623. 10.1016/j.btre.2021.e00623 PMC8120861 34026575

[43] Raper KB, Fennell DI. The genus Aspergillus. 1965.

[44] Nims E, Dubois CP, Roberts SC, Walker EL. Expression profiling of genes involved in paclitaxel biosynthesis for targeted metabolic engineering. Metab Eng. 2006;8:385–94. 16793302 10.1016/j.ymben.2006.04.001

[45] Abdel-Fatah SS, El-Sherbiny GM, Khalaf M, Baz AFE, El-Sayed AS, El-Batal AI. Boosting the anticancer activity of *Aspergillus flavus* “endophyte of jojoba” Taxol via conjugation with gold nanoparticles mediated by γ-irradiation. Appl Biochem Biotechnol. 2022;194:3558–81. 35438406 10.1007/s12010-022-03906-8 PMC9270289

[46] El-Sayed AS, Ali GS. *Aspergillus flavipes* is a novel efficient biocontrol agent of *Phytophthora parasitica*. Biol Control. 2020;140:104072.

[47] Tamura K, Peterson D, Peterson N, Stecher G, Nei M, Kumar S. MEGA5: molecular evolutionary genetics analysis using maximum likelihood, evolutionary distance, and maximum parsimony methods. Mol Biol Evol. 2011;28:2731–9. 21546353 10.1093/molbev/msr121 PMC3203626

[48] Bouqellah NA, El-Sayyad GS, Attia MS. Induction of tomato plant biochemical immune responses by the synthesized zinc oxide nanoparticles against wilt-induced *Fusarium oxysporum*. Int Microbiol. 2023. 10.1007/s10123-023-00404-7. 37491678 10.1007/s10123-023-00404-7

[49] Christensen GD, Simpson WA, Bisno AL, Beachey EH. Adherence of slime-producing strains of *Staphylococcus epidermidis* to smooth surfaces. Infect Immun. 1982;37:318–26. 6179880 10.1128/iai.37.1.318-326.1982 PMC347529

[50] Ansari MA, Khan HM, Khan AA, Cameotra SS, Pal R. Antibiofilm efficacy of silver nanoparticles against biofilm of extended spectrum β-lactamase isolates of *Escherichia coli* and *Klebsiella pneumoniae*. Appl Nanosci. 2014;4:859–68.

[51] Agarwal H, Nakara A, Menon S, Shanmugam V. Eco-friendly synthesis of zinc oxide nanoparticles using Cinnamomum Tamala leaf extract and its promising effect towards the antibacterial activity. J Drug Deliv Sci Technol. 2019;53:101212.

[52] Brownlee K. Probit analysis: a statistical treatment of the sigmoid response curve. JSTOR; 1952.

[53] Krishnakumar P, Varghese M, Joe MG, Rajagopal A, Varghese L. Identification and bioactivities of endophytic fungi from *Lagenandra toxicaria* Dalz. and *Kaempferia rotunda* L. J Appl Biol Biotechnol. 2021;9:117–25.

[54] Rodrigues JC, Lima da Silva W, Ribeiro da Silva D, Maia CR, Santos Goiabeira CV, Figueiredo Chagas HD, et al. Antimicrobial activity of *Aspergillus* sp. from the Amazon biome: isolation of kojic acid. Int J Microbiol. 2022;2022:4010018. 35620355 10.1155/2022/4010018 PMC9129978

[55] Ton That Huu D, Phuong HT, Diem Tran PT, Souvannalath B, Trung HL, Ho DV, et al. Secondary metabolites from the grasshopper-derived entomopathogenic fungus *Aspergillus Tamarii* NL3 and their biological activities. Nat Prod Commun. 2022;17:1934578X221141548.

[56] Bayram O, Krappmann S, Ni M, Bok JW, Helmstaedt K, Valerius O, et al. VelB/VeA/LaeA complex coordinates light signal with fungal development and secondary metabolism. Science. 2008;320:1504–6. 18556559 10.1126/science.1155888

[57] Lotfy MM, Sayed AM, AboulMagd AM, Hassan HM, El Amir D, Abouzid SF, et al. Metabolomic profiling, biological evaluation of *Aspergillus awamori*, the river Nile-derived fungus using epigenetic and OSMAC approaches. RSC Adv. 2021;11:6709–19. 35423214 10.1039/d0ra07578g PMC8694877

[58] Uddin MK, Juraimi AS, Ali ME, Ismail MR. Evaluation of antioxidant properties and mineral composition of purslane (*Portulaca oleracea* L.) at different growth stages. Int J Mol Sci. 2012;13:10257–67. 22949859 10.3390/ijms130810257 PMC3431857

[59] El-Batal AI, Mosallam FM, Ghorab M, Hanora A, Gobara M, Baraka A, et al. Factorial design-optimized and gamma irradiation-assisted fabrication of selenium nanoparticles by chitosan and *Pleurotus ostreatus* fermented fenugreek for a vigorous in vitro effect against carcinoma cells. Int J Biol Macromol. 2020;156:1584–99. 31790741 10.1016/j.ijbiomac.2019.11.210

[60] El-Batal AI, Abd Elkodous M, El-Sayyad GS, Al-Hazmi NE, Gobara M, Baraka A. Gum Arabic polymer-stabilized and gamma rays-assisted synthesis of bimetallic silver-gold nanoparticles: powerful antimicrobial and antibiofilm activities against pathogenic microbes isolated from diabetic foot patients. Int J Biol Macromol. 2020;165:169–86. 32987079 10.1016/j.ijbiomac.2020.09.160

[61] Chudobová D, Cihalova K, Kopel P, Ruttkay-Nedecky B, Vaculovicova M, Krozek R, et al. Antimicrobial nanomaterials in the food industry. Kvasny Prum. 2015;61:51–6.

[62] Wang X, Ramalingam M, Chen G, Ma P, Cui F-Z. Biomimetics: advancing nanobiomaterials and tissue engineering. John Wiley & Sons; 2013.

[63] Munna N, Jamal MS, Rahim A, Islam MK, Shakil AR, Kamruzzaman M. In-depth crystallographic structure and optoelectrical properties of ZnO nanoparticles synthesized by a facile precipitation method. Next Materials. 2025;9:100960.

[64] Poyraz S, Cerkez I, Huang TS, Liu Z, Kang L, Luo J, et al. One-step synthesis and characterization of polyaniline nanofiber/silver nanoparticle composite networks as antibacterial agents. ACS Appl Mater Interfaces. 2014;6:20025–34. 25365660 10.1021/am505571m

[65] Belavi P, Chavan G, Naik L, Somashekar R, Kotnala R. Structural, electrical and magnetic properties of cadmium substituted nickel–copper ferrites. Mater Chem Phys. 2012;132:138–44.

[66] Pal K, Elkodous MA, Mohan MM. CdS nanowires encapsulated liquid crystal in-plane switching of LCD device. J Mater Sci Mater Electron. 2018;29:10301–10.

[67] Nissen M, Förster R, Wieduwilt T, Lorenz A, Jiang S, Hauswald W, et al. Nanoparticle tracking in single‐antiresonant‐element fiber for high‐precision size distribution analysis of mono‐and polydisperse samples. Small. 2022;18:2202024. 10.1002/smll.202202024 35988130

[68] Awed AS, El-Sayyad GS, El-ghandour A, Hameed MFO, Abdel Maksoud MIA, El-Batal AI, et al. Unveiling antimicrobial activity of metal iodide (CuI, AgI, and PbI_2_) nanoparticles: towards biomedical surfaces applications. J Cluster Sci. 2019. 10.1007/s10876-019-01744-z.

[69] Souza TG, Ciminelli VS, Mohallem NDS. A comparison of TEM and DLS methods to characterize size distribution of ceramic nanoparticles. J Phys Conf Ser. 2016;733:012039.

[70] Castro-Longoria E, Vilchis-Nestor AR, Avalos-Borja M. Biosynthesis of silver, gold and bimetallic nanoparticles using the filamentous fungus *Neurospora crassa*. Colloids Surf B Biointerfaces. 2011;83:42–8. 21087843 10.1016/j.colsurfb.2010.10.035

[71] Mohsin M, Jawad M, Yameen MA, Waseem A, Shah SH, Shaikh AJ. An insight into the coating behavior of bimetallic silver and gold core-shell nanoparticles. Plasmonics. 2020. 10.1007/s11468-020-01166-y.

[72] Soni J, Revathi D, Dhanraj G, Ramasubburayan R. Bioinspired green synthesis of ZnO nanoparticles by marine-derived *Streptomyces plicatus* and its multifaceted biomedicinal properties. Microb Pathog. 2024;193:106758. 38906493 10.1016/j.micpath.2024.106758

[73] Imath M, Giri J, Mohammad F, Ragavendran C. Eco-friendly synthesis of ZnO nanoparticles fabricated using *Fioria vitifolia* L. and their biomedical potentials. Microb Pathog. 2025;199:107139. 39579945 10.1016/j.micpath.2024.107139

[74] Shakerdarabad R, Mohabatkar H, Behbahani M, Dini G. Antibiofilm and antibacterial activities of green synthesized ZnO nanoparticles against *Erwinia amylovora* and *Pseudomonas syringae* pv. *Syringae*: in vitro and in silico investigations. Microb Pathog. 2024;196:107011. 39396688 10.1016/j.micpath.2024.107011

[75] Velázquez-Hernández AM, Martínez-Gallegos S, Albiter V, González-Juárez J, García-Ibarra C. Synthesis of an antimicrobial chitosan film impregnated with ZnO nanoparticles phytosynthesized with *Ruta graveolens* plant extract. Microb Pathog. 2025;200:107268. 39742899 10.1016/j.micpath.2024.107268

[76] Baptista PV, McCusker MP, Carvalho A, Ferreira DA, Mohan NM, Martins M, et al. Nano-strategies to fight multidrug resistant bacteria—“A Battle of the Titans.” Front Microbiol. 2018;9:1441. 30013539 10.3389/fmicb.2018.01441 PMC6036605

[77] Arora N, Thangavelu K, Karanikolos GN. Bimetallic nanoparticles for antimicrobial applications. Front Chem. 2020;8:412. 32671014 10.3389/fchem.2020.00412 PMC7326054

[78] Zhang L, Jiang Y, Ding Y, Povey M, York D. Investigation into the antibacterial behaviour of suspensions of ZnO nanoparticles (ZnO nanofluids). J Nanopart Res. 2007;9:479–89.

[79] Xia Y, Zhou Y, Tang Z. Chiral inorganic nanoparticles: origin, optical properties and bioapplications. Nanoscale. 2011;3:1374–82. 21301709 10.1039/c0nr00903b

[80] Hashem AH, Saied E, Ali OM, Selim S, Al Jaouni SK, Elkady FM, et al. Pomegranate peel extract stabilized selenium nanoparticles synthesis: promising antimicrobial potential, antioxidant activity, biocompatibility, and hemocompatibility. Appl Biochem Biotechnol. 2023. 10.1007/s12010-023-04326-y. 36705842 10.1007/s12010-023-04326-y

[81] Dryden M. Reactive oxygen species: a novel antimicrobial. Int J Antimicrob Agents. 2018;51:299–303. 28887201 10.1016/j.ijantimicag.2017.08.029

[82] Hashem AH, El-Sayyad GS. Antimicrobial and anticancer activities of biosynthesized bimetallic silver-zinc oxide nanoparticles (Ag-ZnO NPs) using pomegranate peel extract. Biomass Convers Biorefinery. 2023. 10.1007/s13399-023-04126-8.

[83] Nawaz A, Farhan A, Maqbool F, Ahmad H, Qayyum W, Ghazy E, et al. Zinc oxide nanoparticles: pathways to micropollutant adsorption, dye removal, and antibacterial actions-a study of mechanisms, challenges, and future prospects. J Mol Struct. 2024. 10.1016/j.molstruc.2024.138545.

[84] Priyadarshini E, Rawat K, Bohidar HB. Quantum dots-based nano-coatings for inhibition of microbial biofilms: a mini review. In: Nonmagnetic and magnetic quantum dots. IntechOpen; 2017.

[85] Shafiey SI, Ahmed KA, Abo-Saif AA, Abo-Youssef AM, Mohamed WR. Galantamine mitigates testicular injury and disturbed spermatogenesis in adjuvant arthritic rats via modulating apoptosis, inflammatory signals, and IL-6/JAK/STAT3/SOCS3 signaling. Inflammopharmacology. 2024;32:405–18. 37429998 10.1007/s10787-023-01268-z PMC10907493

[86] Rajesh S, Dharanishanthi V, Kanna AV. Antibacterial mechanism of biogenic silver nanoparticles of *Lactobacillus acidophilus*. J Exp Nanosci. 2015;10:1143–52.

[87] Azam Z, Ayaz A, Younas M, Qureshi Z, Arshad B, Zaman W, et al. Microbial synthesized cadmium oxide nanoparticles induce oxidative stress and protein leakage in bacterial cells. Microb Pathog. 2020;144:104188. 32272217 10.1016/j.micpath.2020.104188

[88] Paul D, Maiti S, Sethi DP, Neogi S. Bi-functional NiO-ZnO nanocomposite: synthesis, characterization, antibacterial and photo assisted degradation study. Adv Powder Technol. 2021;32:131–43.

