# Supplementary material for: Endophytic fungi-assisted biological synthesis of zinc oxide nanoparticles using gamma-rays for promising antibacterial and antibiofilm potential against some gram-positive bacteria
Source: Microb Cell Fact. 2026 Apr 20;25:106. doi: 10.1186/s12934-026-02993-0 (PMC13097906; doi:10.1186/s12934-026-02993-0)
Supplement: Supplementary file 1 — Additional file 1. [file 12934_2026_2993_MOESM1_ESM.docx]

***
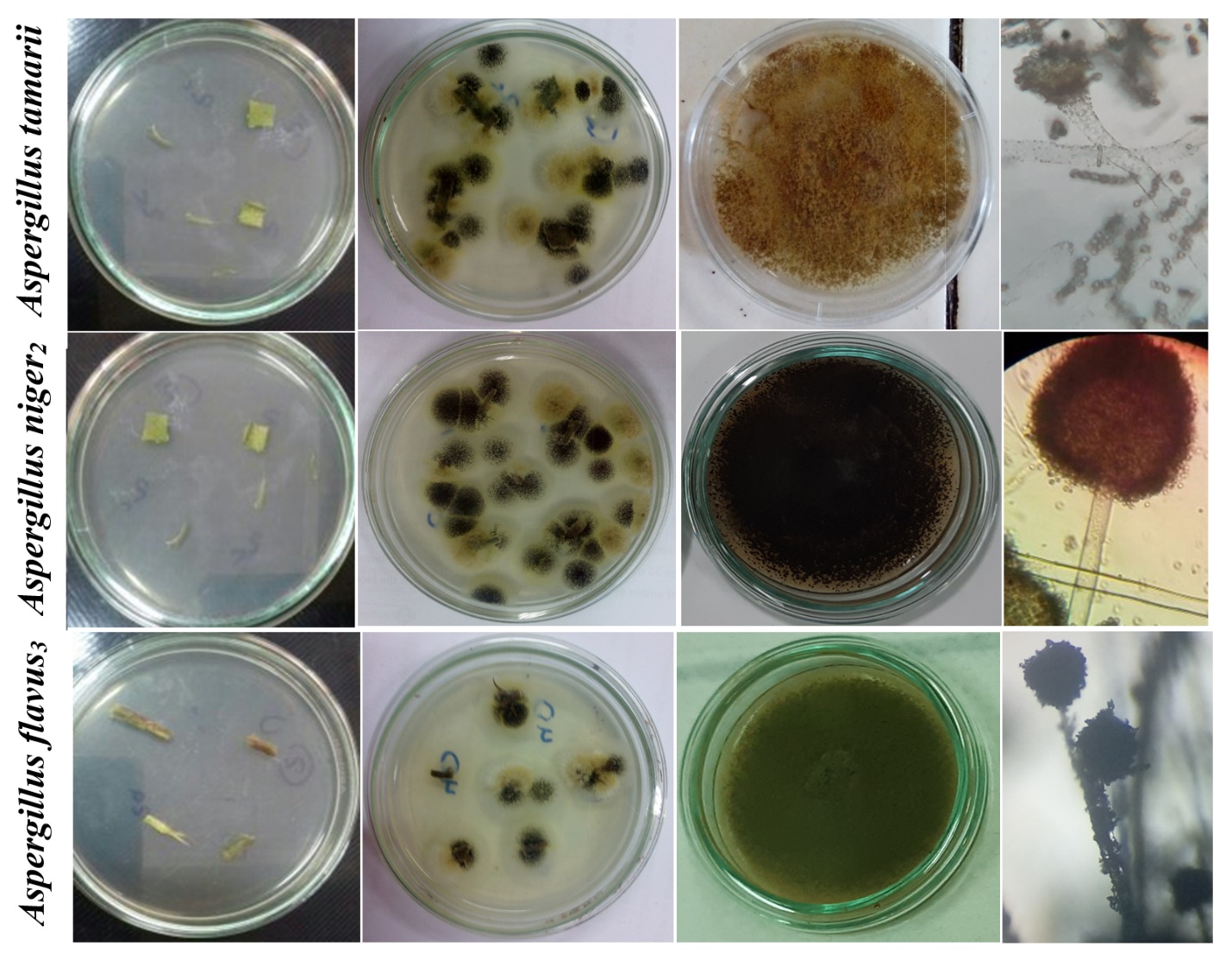
***

**Figure (S1): Isolation, purification and microscopic examination of some endophytic fungi from leaves and stem of (*Ocimum basilicum*).**
